# Dynamic spectrum sharing between active and passive users above 100 GHz

**DOI:** 10.1038/s44172-022-00002-x

**Published:** 2022-05-26

**Authors:** Michele Polese, Viduneth Ariyarathna, Priyangshu Sen, Jose V. Siles, Francesco Restuccia, Tommaso Melodia, Josep M. Jornet

**Affiliations:** 1 Institute for the Wireless Internet of Things, Northeastern University, Boston, MA USA; 2 Jet Propulsion Laboratory, California Institute of Technology, Pasadena, CA USA; 3 Roux Institute, Northeastern University, Portland, ME USA

**Keywords:** Electrical and electronic engineering, Policy, Information technology

## Abstract

Sixth-generation wireless networks will aggregate higher-than-ever mobile traffic into ultra-high capacity backhaul links, which could be deployed on the largely untapped spectrum above 100 GHz. Current regulations however prevent the allocation of large contiguous bands for communications at these frequencies, since several narrow bands are reserved to protect passive sensing services. These include radio astronomy and Earth exploration satellites using sensors that suffer from harmful interference from active transmitters. Here we show that active and passive spectrum sharing above 100 GHz is feasible by introducing and experimentally evaluating a real-time, dual-band backhaul prototype that tracks the presence of passive users (in this case the NASA satellite Aura) and avoids interference by automatically switching bands (123.5–140 GHz and 210–225 GHz). Our system enables wide-band transmissions in the above-100-GHz spectrum, while avoiding harmful interference to satellite systems, paving the way for innovative spectrum policy and technologies in these crucial bands.

## Abstract (web summary)

Michele Polese and colleagues report a dual-band backhaul prototype operating above 100 GHz. It can be dynamically re-configured in real time to avoid interference to passive satellite sensing systems, enabling safe coexistence of active and passive users in this portion of the spectrum.

## Introduction

The digital transformation of our society is fostered by the availability of a fundamental, invisible, yet scarce, resource – the electromagnetic spectrum^1–4^. Besides enabling information exchange through wireless communications, the electromagnetic spectrum is also a rich source of information through sensing. The finite nature of the spectrum creates competing interests for communications and sensing. These diverging interests, expressed by different scientific communities, government entities, and industries, have led to rigid spectrum allocations from national and international regulatory bodies, such as the International Telecommunications Union (ITU)^5^, or the Federal Communications Commission (FCC)^6^, dating back to the 1930s.

To support more devices and ultra-high capacity applications, 6th Generation (6G) wireless networks will require data rates that are orders of magnitude higher than available today, thus boosting the need for spectrum^7^. While the 5th Generation (5G) of mobile networks uses carrier frequencies as high as 71 GHz^8^, 6G will move beyond 100 GHz^9–11^ to aggregate data of many mobile users in ultra-high capacity backhaul links.

However, communications in this spectrum band are limited by the coexistence of passive users that (i) do not transmit and (ii) only use high-sensitivity Radio Frequency (RF) sensors for Earth exploration, weather monitoring and radio-astronomy^6,12^. Passive users can be negatively impacted by interference from active transmissions^13^. Therefore, they retain exclusive access to relatively narrow portions of the above-100-GHz spectrum, preventing the allocation of contiguous chunks with tens of GHz of bandwidth for communications^5,6^. For example, in the U.S., the largest allocations for active transmissions between 100 and 275 GHz are 32.5 GHz (116−148.5 GHz) and 18.5 GHz (231.5−250 GHz), but with only 12.25 GHz (non contiguous) earmarked for unrestricted fixed or mobile terrestrial usage^6^. Transmissions are strictly forbidden in 33.5 GHz of spectrum, and conditioned to the protection of coexisting passive users in the remaining spectrum.

These conservative regulations apply even without passive users exploiting the spectrum for sensing. This prevents multiplexing of unused resources, making this spectrum less appealing for wireless backhaul^14^. To foster wireless innovation in years to come, it becomes necessary to develop spectrum sharing solutions between communications and passive sensing systems – which is the main contribution of this paper. Moreover, while terrestrial sensing stations can be protected through geographical separation, orbiting satellite systems call for dynamic sharing solutions based on self-adaptive wireless links. Indeed, we show—through an accurate link budget based on ITU channel models—that active transmitters may actually generate harmful interference to highly sensitive sensors on satellites orbiting over communication systems.

Shared access between sensing and communications has never been demonstrated experimentally in the spectrum above 100 GHz. This is not without reason. Current spectrum policies prohibit transmissions in passive bands, even for experimental purposes. Additionally, a self-adaptive wireless link requires the design of dynamic sub-terahertz RF front-ends and protocol stacks that need to be controlled and reconfigured in real time. However, existing transceivers designed for this portion of the spectrum are limited to channel sounding or static baseband processing capabilities, with limited or no reconfigurability, and are thus unfit to demonstrate dynamic spectrum access^15–30^. This paper presents a dynamic spectrum access, dual-band backhaul system in the above-100-GHz spectrum, specifically, at 123.5–140 GHz (referred to as Lower Band (LB) throughout the paper) and 210–225 GHz (Upper Band (UB)). This is the first above-100-GHz wireless system that (i) operates a real-time protocol stack in two frequency bands; (ii) performs automated and dynamic spectrum sharing; and (iii) adapts link parameters (e.g., the frequency band) in real time. The proposed system tracks satellite orbits and automatically switches frequency bands, thus preventing backhaul transmissions from interfering with passive incumbents, while enabling the allocation of large, contiguous bands for communication services on shared spectrum. Prototyping this system has required us to address challenges that span from RF design for the dual-band setup, to dynamic control and integration of multiple independent systems through open interfaces. We evaluate the backhaul prototype in an experimental setting with transmitter and receiver deployed outdoor in an urban environment, on the roofs of two buildings, under the limits of an FCC experimental license. We believe that the results shown in this paper can pave the way for more flexible regulations, protecting scientific uses of the spectrum while allowing more reasonable allocations for future generation of communication systems.

## Results

This section introduces the proposed self-adaptive dual-band backhaul link. We discuss the challenges and lessons learned in the development of the RF and communication components, the dynamic switching framework, and present its experimental performance evaluation.

### RF and communications components

**Fig. 1:**
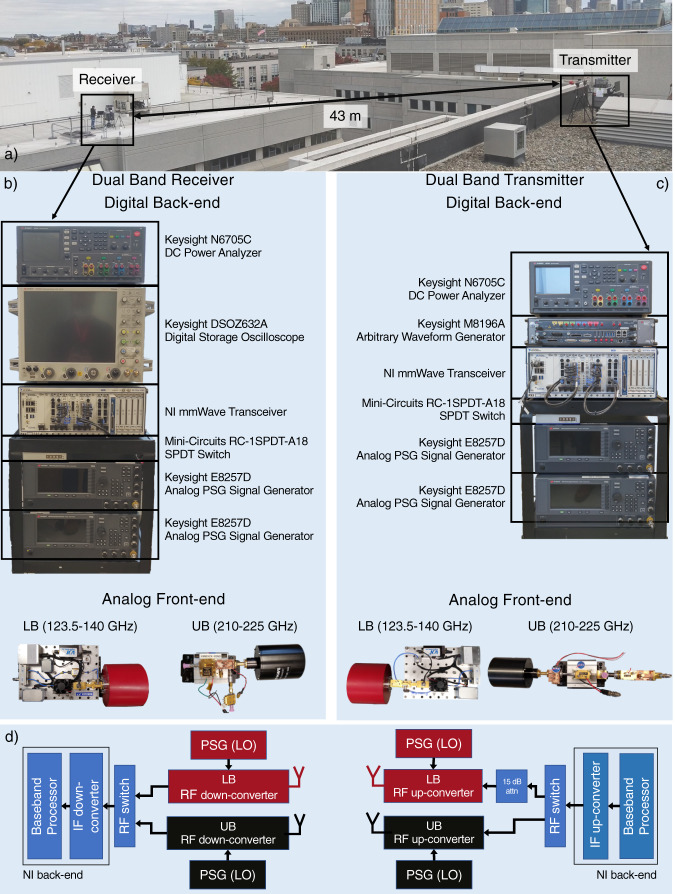
Prototype schematics and dual-band backhaul link deployment. **a** Deployment of the dual-band backhaul link over the Snell Engineering and Egan Research buildings at Northeastern University. **b** Components of the RX. **c** Components of the TX. **d** Block diagram with the components of the TX and RX. DC stands for Direct Current, NI for National Instruments, SPDT for Single-Pole Double-Throw, PSG for Performance Signal Generator, LB for Lower Band (123.5–140 GHz), UB for Upper Band (210–225 GHz), LO for Local Oscillator, IF for Intermediate Frequency, and RF for Radio Frequency.

Figure 1 shows the prototype hardware, with Transmitter (TX)/Receiver (RX) real-time signal processing back-ends and dual-band sub-terahertz front-ends. The NI (formerly known as National Instruments) mmWave back-end^31^ features a hardware-accelerated protocol stack that generates an Orthogonal Frequency Division Multiplexing (OFDM) waveform inspired by the 5G New Radio (NR) physical (PHY) layer, aggregating eight component carriers with 100 MHz each^32^. The dual-band front-ends, supporting 123.5–140 GHz and 210–225 GHz, accept an Intermediate Frequency (IF) input at the up-converters (TX-side) and generate an IF output at the down-converters (RX-side). They are connected to the baseband-to-IF conversion modules of the NI system through a Single-Pole Double-Throw (SPDT) RF switch, which supports DC–18 GHz^33^. Four Keysight E8257D Performance Signal Generators (PSGs) drive the Local Oscillators (LOs) of the dual-band front-ends.

The overall system design was hindered by two main challenges. First, the same IF output, with high peak-to-average power ratio, needs to be interfaced with dual-band front-ends with different and limited input power specifications. Second, phase noise and dual sideband front-ends introduce amplitude impairments in the received signal.

The link budget planning aims at having a 10 decibel (dB) SNR margin at the IF input of the receiver back-end, independently on the LB or UB front-ends. This is challenged by two factors. First, the back-end OFDM implementation introduces a high peak-to-average power ratio (>10 dB), even for the lowest order modulations. This is a known drawback for OFDM systems using large Fast Fourier Transform (FFT) sizes (2048 for each component carrier, in this case). Second, there is an input power mismatch between the LB and UB TX front-ends. The LB front-end needs an input signal with power lower than 0 decibel-milliwatts (dBm), and generates a maximum 13 dBm output power. The UB front-end needs an input power of 10 dBm to generate a 3 dBm output at the desired harmonic. To address this, we precisely characterized the OFDM waveform power, and reduced the back-end output power to maintain the peak power under the limit of the UB front-end. The input to the LB front-end was then further attenuated to be below 0 dB. Finally, to close the link margin for the 43 m link, the LB and UB systems use 38 dBi and 40 dBi antennas, respectively.

The TX and RX at both bands feature two mixing stages, part of the analog front-ends shown in Fig. 1, thus forming a heterodyne system. However, due to the lack of RF filters at the considered frequency ranges, both LB and UB front-ends lack a sideband selection filter. This, combined with the high phase noise (due to the frequency multiplication process), may lead to drastic amplitude fluctuations in the received signal. To address this, we offset the IF frequency in the up- and down-converting paths by 1 GHz, different from traditional heterodyne systems, which use the same IF frequencies. Thus, only one of the sidebands is selected for receiver-side processing, preventing undesired amplitude fluctuations. To enable quick dynamic switching, the LB and UB front-ends are set to the same IF frequency.

The frequency offset is compensated by appropriately setting the IF-to-baseband and the IF-to-RF LO frequencies in the NI back-end and the two LB and UB transmit-receive paths. Notably, the LB front-ends perform a 4 × frequency multiplication of the LO input, thus the TX LO is set to 32.5 GHz (130 GHz at the up-converter), while the RX LO is set to 32.75 GHz (131 GHz at the down-converter). Similarly, UB converters utilize an 9× (6×) LO multiplication at the TX (RX) side. Therefore, the LO input at the TX-side was set to 23.88 GHz (corresponding to 215 GHz) and RX-side was set to 216/6 = 36 GHz, selecting the upper sideband for baseband processing.

### Dynamic frequency switching framework

The dynamic control of the dual-band front-end requires the integration of the hardware components with a programmable, software-based control logic that can automatically switch bands when the active system interferes with a passive incumbent. The framework features (i) a tracking system that identifies when LB or UB transmissions may be interfering with a passive user (e.g., a satellite orbiting over the link); and (ii) an interface with the SPDT switch.

This flexible architecture can operate in two different modes. The first one is named independent switching, and assumes no coordination between the backhaul endpoints, which are only loosely time-synchronized to a common source through the Network Time Protocol (NTP) and perform frequency switching decisions independently. This configuration represents an ad hoc network deployment, without out-of-band control. The framework runs on the NI back-ends. We call the second deployment mode coordinated switching. It mimics the configuration of backhaul nodes for cellular networks, where the equipment is connected to a control overlay^34^. The tracking framework is hosted by a central controller, which notifies the two endpoints simultaneously.

We considered as passive user the NASA satellite Aura, which studies the Earth climate and air quality^35^ and is listed as incumbent in the spectrum adjacent to the UB band in the World Meteorological Organization (WMO) Observing Systems Capability Analysis and Review Tool (OSCAR) database^36^. Note that we operate the UB backhaul link in the 210–225 GHz range, compliant with FCC regulations and an experimental license. However, its RF front-end could extend up to 240 GHz, thus potentially interfering with the Aura Microwave Limb Scanner (MLS). This instruments scans the ozone (O_3_) absorption line around 235.7098 GHz, and the carbon monoxide (CO) line at 230.538 GHz^12^. Therefore, in this specific setup, we consider LB as the band where UB traffic can be offloaded to avoid interference to the Aura MLS. The tracking system retrieves Aura’s orbits from public Application Programming Interfaces (APIs)^37^ and checks whether the satellite is orbiting over the backhaul link deployment. Harmful interference is detected when the satellite and backhaul are in Line-of-Sight (LOS), or the actual link budget exceeds an ITU threshold (as discussed next). Accordingly, the SPDT switch interface triggers an UB-to-LB band switch through HyperText Transfer Protocol (HTTP) APIs^38^. The switching mechanism can be further refined to avoid unnecessary or too frequent band switching that may degrade the system performance.

### Experimental evaluation

The dual-band transmitter and receiver were deployed outdoor, on top of two Northeastern University campus buildings, at a distance of 43 m, as shown in Fig. 1.

**Fig. 2:**
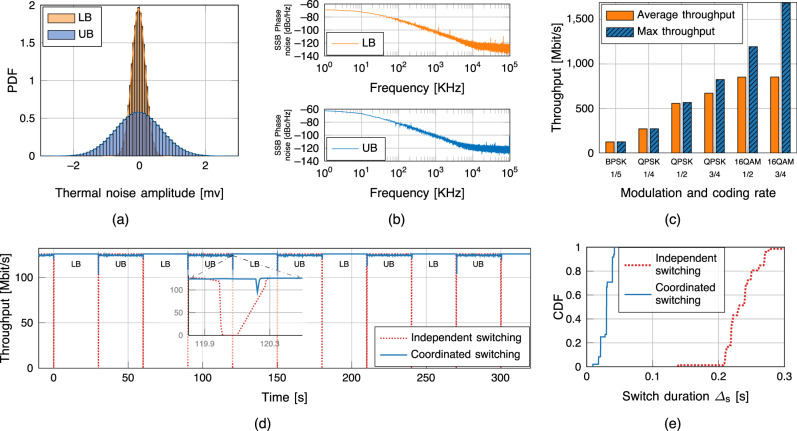
Experimental result for the dual-band self-adaptive backhaul link. The LB front-end operates in the 123.5–140 GHz band (and, in this specific case, at 139 GHz), and the UB front-end operates in the 210–225 GHz band (specifically, at 224 GHz). **a** Probability distribution function (PDF) of the thermal noise amplitude for the LB (orange histogram) and UB (blue histogram) front-ends of the dual-band system. Analysis based on *n* = 3.2 × 10^6^ noise samples. **b** Single Sideband (SSB) phase noise for the LB (orange line) and UB (blue line) front-ends. Analysis based on *n* = 1.28 × 10^9^ noise samples. dBc is decibel relative to the carrier. **c** Average (orange bars) and maximum (blue bars) throughput for different Modulation and Coding Schemes (MCSs). Analysis based on *n* = 215,062 throughput samples. Mbit/s is Megabit per second. **d** Throughput over time for two independent experiments with MCS Binary Phase Shift Keying (BPSK) 1/5, with independent (dotted red line) and coordinated (solid blue line) band switching. **e** Cumulative distribution function (CDF) of duration Δ_s_ of a band switch procedure for independent switching (red line) and coordinated switching (blue line). Analysis based on *n* = 120 switching events.

Despite the similar link budget, the higher thermal and Single Sideband (SSB) phase noise (Fig. 2a, b) of the UB front-end (with  a ~ 10 dB noise figure increase) affects its throughput when higher Modulation and Coding Schemes (MCSs) are considered (Fig. 2c). The maximum throughput (achieved with the LB system) and the average throughput (which accounts for both) are comparable up to a Quadrature Phase Shift Keying (QPSK) modulation with a 1/2 coding rate. Less robust MCSs lead to lower throughput for UB. As a consequence, the average throughput is below the maximum achievable by the system.

To evaluate the effectiveness of the proposed frequency switching approach, Fig. 2d reports the throughput over time with independent and coordinated switching artificially triggered every 30 s. The system maintains stable throughput with negligible impact of the switching procedure, especially with coordinated switching. This is only an illustrative example to show the effectiveness of the switching mechanism, as the actual time spent in each frequency band varies and depends on the presence of passive users in the UB band. Figure 2f plots the cumulative distribution function of the switching duration Δ_s_ over 120 independent switching events, defined as the time interval between the switch at one endpoint and the switch at the other. Independent switching requires a median value of Δ_s_ = 230 ms, as the switch may happen at slightly different time instants in the two endpoints, which only rely on NTP for a loose synchronization^39^. The coordinated approach takes <42 ms in the worst case (median 30.11 ms), as the switch is triggered by a centralized controller for both endpoints. A short switching time also allows the system to easily scale in case multiple incumbents need to be tracked and avoided. Overall, independent switching—which facilitates deployment in challenging environments—has a trade off in performance with coordinated switching. Even in case the frequency switch happens infrequently, and thus has limited impact on the average throughput, a downtime of tens of milliseconds may affect the end-to-end performance of traffic flows traversing the link, e.g., it may trigger retransmissions or session resets at the transport or application layers. Nonetheless, both switching mechanisms allow the prototype to promptly avoid harmful interference, while maintaining high average throughput values.

## Discussion

**Fig. 3:**
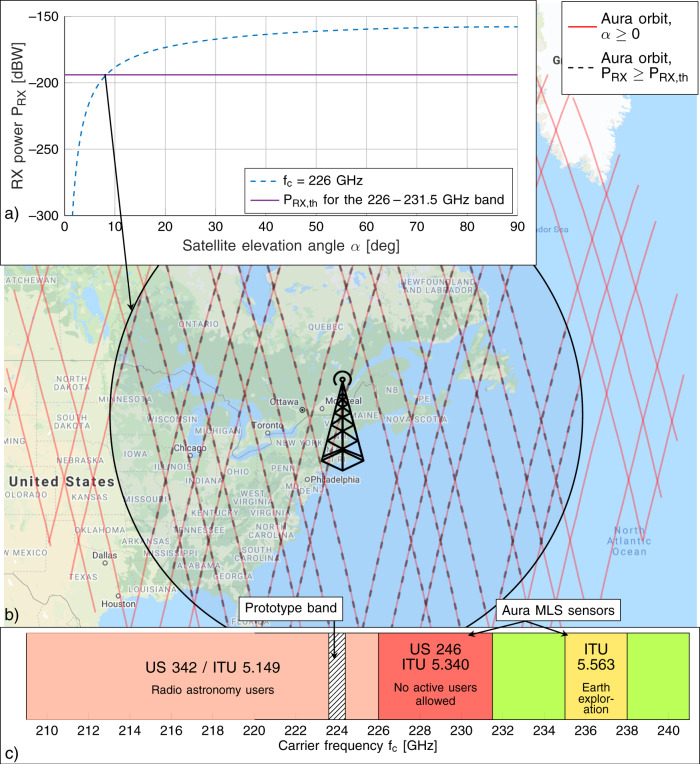
Link budget analysis, aura satellite orbits, and spectrum. **a** Received (RX) power *P*_RX_ (blue dashed line) for a satellite on the Aura orbit from a ground transmitter operating at *f*_c_ = 226 GHz, compared with the ITU threshold for interference *P*_RX,th_ (solid purple line), in decibel-watts (dBW). **b** Aura satellite orbit over the prototype deployment location, with elevation angle *α* ≥ 0 (solid red lines) and with elevation angle that corresponds to *P*_RX_ ≥ *P*_RX,th_ (black dashed line). **c** Summary of spectrum regulations for the 209–241 GHz band. ITU 5.340 bands, or United States (US) 246 (226–231.5 GHz), are represented in red; ITU 5.149 bands, or US 342 (209–226 GHz), in orange; ITU 5.563 bands (235–238 GHz) in yellow; and bands in which communication users are (co-)primary (231.5–235 GHz and 238–241 GHz) in green. The band of the prototype is shown in white with black solid lines. MLS is Microwave Limb Scanner.

To understand the dynamic spectrum sharing opportunities above 100 GHz (specifically, between 209–241 GHz), Fig. 3 illustrates spectrum regulations (bottom)^5,6^, and a link budget for the worst-case interference toward a sensing system on the Aura satellite orbiting over the backhaul link (top). For this analysis, we focus on the 209–241 GHz band, as it is spectrum in which the UB front-end operates, but similar coexistence issues apply to the ITU-regulated spectrum above 100 GHz^5^.

The 209–241 GHz spectrum includes bands where active users (i) cannot transmit, (226–231.5 GHz, according to footnotes FCC US246^6^ and ITU 5.340^5^); or (ii) need to protect passive radio-astronomy and Earth monitoring users (209–226 GHz and 235–238 GHz, according to FCC US342, ITU 5.149, and 5.563). The ITU defines harmful interference as received power above the threshold *P*_RX,th_ = − 194 dBW in the 226 − 231.5 GHz band^40^. The top left corner of Fig. 3 evaluates whether this threshold is exceeded for Aura—which orbits 705 km over the Earth^35^—by reporting the received power *P*_RX_ for different elevation angles *α*. We conservatively assume that the backhaul link directional antenna (with a 40 dB gain, and 23 dBm of transmit power) steers toward the satellite in a LOS scenario. This is usually not the case, yet provides a worst-case estimate of the interference. We consider the ITU channel models for the atmospheric attenuation as a function of frequency, height, and elevation angle *α* (refs. ^41,42^). The carrier frequency is set to *f*_*c*_ = 226 GHz, i.e., the closest frequency to the UB carrier actually used in the experiment (224 GHz) with a threshold defined in ref. ^40^. While the atmospheric attenuation of the signal prevents interference for small *α*, for *α* ≥ 8.4° the received power exceeds *P*_RX,th_. This translates into satellite passes (highlighted in Fig. 3) for which the dual-band backhaul link needs to switch from UB to LB.

**Fig. 4:**
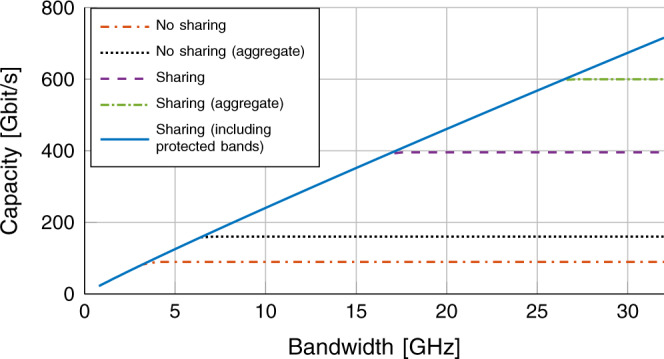
Capacity in Gigabit per second (Gbit/s) as a function of the bandwidth *B*. The analysis assumes a received (RX) power *P*_RX_ = − 23 dBm, a noise figure *F* = 8.5 dB for the receiver, a noise temperature *T*_0_ = 296 K. The configuration “No sharing” only uses bands which are exclusively for fixed or mobile communications, without (red dash dotted line) or with aggregation (black dotted line). The “Sharing” option leverages the bands in which communications are primary or co-primary, without (purple dashed line) or with aggregation (green dash dotted line). The “Sharing (including protected bands)” setup (blue solid line) also includes sharing of ITU 5.340 bands.

This considered, sharing solutions that would enable contiguous, large bandwidth transmissions in the above-100-GHz bands must not be static. Dynamic spectrum sharing would allow regulations to evolve from a firm prohibition of any active transmissions in passive bands, to more flexible schemes where active users could transmit on ultra-large contiguous bandwidths for a portion of time. For example, by considering the 209−241 GHz band, only 6.5 GHz (non contiguous, 231.5−235 GHz and 238−241 GHz) are earmarked for fixed or mobile transmission without the need to share spectrum and thus protect passive incumbents. Figure 4 reports the Shannon capacity as a function of the bandwidth *B* for different spectrum sharing and aggregation configurations. We consider a received power *P*_RX_ = − 23 dBm, a noise figure *F* = 8.5 dB for the receiver, a noise temperature *T*_0_ = 296 K. The Signal-to-Noise-Ratio (SNR) in dB is ${{\Gamma }}={P}_{{{{{{{{\rm{RX}}}}}}}}}-10{\log }_{10}(k{T}_{0}B)-F$, and the Shannon capacity is computed as $C=B{\log }_{2}(1+{{\Gamma }})$. According to current regulations, we limit the capacity to the value supported by the maximum bandwidth allowed by the FCC allocations (e.g., 3.5 GHz for the “No sharing” configuration). We also consider the possibility of aggregating multiple frequency bands for a single transmission, in this case the “No sharing” configuration would exploit 3.5 + 3 = 6.5 GHz. Note that carrier aggregation may not be feasible for all wireless communication technologies. Figure 4 clearly shows the gap in capacity between a system where dynamic spectrum sharing is allowed and one which follows the current regulations, with a gain of up to 8x (no aggregation) and 4.5x (aggregation). Additionally, results in Polese et al.^43^ show that, to reach a target rate of 1 Terabit per second (Tbit/s), future 6G systems above 100 GHz will require more contiguous bandwidth than what is available without spectrum sharing.

Enabling dynamic spectrum sharing requires (i) awareness of passive users impacted by interference; and (ii) self-adaptive active links. In this paper, we show a possible approach toward gathering awareness of operations of passive sensing instruments in specific frequency, time, and geographical locations. This demonstrates that coordination is indeed feasible, and that scalable systems similar to the Spectrum Access System (SAS) in the Citizens Broadband Radio Service (CBRS) band can be introduced to share information on passive sensing activities and plan interference-free operations with active users^43,44^.

The results of this paper also illustrate how our prototype is capable of self-configuring in real-time in these frequency bands. Prior literature has shown wireless systems above 100 GHz^45^, but only as channel sounders^15–18^ or PHY implementations^19–30^. This paper evolves the state of the art on above-100-GHz communications by introducing dynamic control and dual-band operations. While this prototype focuses on real-time adaptability, its back-end supports a limited bandwidth (i.e., 800 MHz), due to constraints in the Analog to Digital Converter (ADC)/Digital to Analog Converter (DAC) rate and signal processing capabilities of its digital fabrics. Therefore, we are also developing a software-defined back-end for multi-GHz-wide transmissions and higher data rates^46^. Nonetheless, the development of a dynamic and real-time re-configurable sub-terahertz link represents a key step to fully unleash the potential of above-100-GHz communications^11^. Next-generation cellular networks will have much higher deployment density^47^, increasing the number of sub-terahertz devices and making manual intervention and configuration close to impossible. A self-organizing link improves the resiliency of such deployments. Besides switching operating bands, above-100-GHz backhaul links need to automatically adapt to atmospheric conditions, including humidity and wind. Humidity affects the wide-band frequency response^48^, increasing the molecular absorption (and thus the path loss) selectively over the electromagnetic spectrum. Consequently, wireless links need to select the appropriate bandwidth or use multi-carrier systems with adaptive MCS, carrying more information on carriers less affected by absorption. Wind, instead, may impact directional transmissions by introducing vibrations in the radio infrastructure^49^.

## Conclusions

Today, the spectrum above 100 GHz is rigidly regulated and highly fragmented, with several narrow bands reserved for passive users. This prevents communication systems from exploiting ultra-wide bandwidths. This paper has demonstrated the feasibility of dynamic spectrum sharing in the above-100-GHz spectrum. We have introduced a dynamic, dual-band backhaul link, showcased real-time adaptability of an above-100-GHz wireless link, and discussed how dynamic spectrum sharing benefits the development of wireless communications systems in these frequency bands. This fundamental result demonstrates how future regulations for this spectrum band may rely on sharing technologies to guarantee seamless coexistence of passive and active users, and fosters future research on design of passive/active awareness and coexistence schemes in the spectrum above 100 GHz.

## Methods

This section reviews relevant information related to the dual-band backhaul link development and its experimental evaluation. The selection of the appropriate LO frequencies and a balanced link budget between the two frequency bands were fundamental to design a high-throughput dual-band backhaul link. In this regard, we first discuss in details how the LO frequency selection can prevent amplitude modulation due to phase noise, and then report the link budget evaluation. Next, we present the scenario and procedures for the experimental evaluation, and introduce additional elements on how the performance metrics were defined and measured. Finally, we review the channel model used to compute the interference to the Aura MLS sensor.

### Frequency planning

We have carefully selected the TX- and RX-side LO (both baseband-to-IF and IF-to-RF) frequencies to mitigate the phase noise contaminating the received signal. The transmitted signal *s*(*t*) from the front-ends of the prototype can be expressed as follows, as both transmitters do not incorporate a bandpass filter to suppress sideband transmissions:

1 $$s(t)=\cos \left({\omega }_{{{{{{{{{\rm{LO}}}}}}}}}_{{{{{{{{\rm{TX}}}}}}}}}}t+{\phi }_{{{{{{{{\rm{TX}}}}}}}}}\right){{{{{{{\rm{Re}}}}}}}}\{p(t){e}^{-j{\omega }_{{{{{{{{\rm{IF}}}}}}}}}t}\},$$

where *p*(*t*) is the complex baseband signal, ${\omega }_{{{{{{{{{\rm{LO}}}}}}}}}_{{{{{{{{\rm{TX}}}}}}}}}}$ and *ω*_IF_ are the frequencies of the IF-to-RF and baseband-to-IF LOs, respectively, *ϕ*_TX_ is the phase noise term at the TX side, and ${{{{{{{\rm{Re}}}}}}}}\{\cdot \}$ denotes the selection of the real part of the signal.

Assuming noiseless and LOS propagation, the received signal *r*(*t*) after the first IF stage can be expressed as

2 $$r(t)=	\;k\;\cdot LPF\left\{\cos \left({\omega }_{{{{{{{{{\rm{LO}}}}}}}}}_{{{{{{{{\rm{TX}}}}}}}}}}t+{\phi }_{{{{{{{{\rm{TX}}}}}}}}}\right)\cdot {{{{{{{\rm{Re}}}}}}}}\left\{p(t){e}^{-j{\omega }_{{{{{{{{\rm{IF}}}}}}}}}t}\right\}\right.\\ 	\cdot \cos \left.\left({\omega }_{{{{{{{{{\rm{LO}}}}}}}}}_{{{{{{{{\rm{RX}}}}}}}}}}t+{\phi }_{{{{{{{{\rm{RX}}}}}}}}}\right)\right\},$$

where *k* accounts for the losses, *LPF*{ ⋅ } denotes the low pass filtering operation, ${\omega }_{{{{{{{{{\rm{LO}}}}}}}}}_{{{{{{{{\rm{RX}}}}}}}}}}$ is the RF-to-IF LO frequency, and *ϕ*_RX_ is the phase noise term at the RX side. The above expression can be simplified to the following form:

3 $$r(t)=	\; k/2\;\cdot\, \cos \left(({\omega }_{{{{{{{{{\rm{LO}}}}}}}}}_{{{{{{{{\rm{TX}}}}}}}}}}-{\omega }_{{{{{{{{{\rm{LO}}}}}}}}}_{{{{{{{{\rm{RX}}}}}}}}}})t+{\phi }_{{{{{{{{\rm{TX}}}}}}}}}-{\phi }_{{{{{{{{\rm{RX}}}}}}}}}\right)\\ 	\cdot {{{{{{{\rm{Re}}}}}}}}\{p(t){e}^{-j{\omega }_{{{{{{{{\rm{IF}}}}}}}}}t}\}.$$

Eq. (3) remarks that when ${\omega }_{{{{{{{{{\rm{LO}}}}}}}}}_{{{{{{{{\rm{TX}}}}}}}}}}={\omega }_{{{{{{{{{\rm{LO}}}}}}}}}_{{{{{{{{\rm{RX}}}}}}}}}}$, the desired received IF signal ${{{{{{{\rm{Re}}}}}}}}\{p(t){e}^{-j{\omega }_{{{{{{{{\rm{IF}}}}}}}}}t}\}$ is being amplitude-modulated by the term $\cos ({\phi }_{{{{{{{{\rm{TX}}}}}}}}}-{\phi }_{{{{{{{{\rm{RX}}}}}}}}})$. Since both *ϕ*_TX_ and *ϕ*_RX_ are time-dependent due to phase noise, the above modulation can cause considerable signal level fluctuation at the received signal at the first IF stage, and there onward down the RX chain. We design our prototype with ${\omega }_{{{{{{{{{\rm{LO}}}}}}}}}_{{{{{{{{\rm{TX}}}}}}}}}}\,\ne\, {\omega }_{{{{{{{{{\rm{LO}}}}}}}}}_{{{{{{{{\rm{RX}}}}}}}}}}$. For this scenario, as seen in Eq. (3), the desired spectrum is centered at $| {\omega }_{{{{{{{{{\rm{LO}}}}}}}}}_{{{{{{{{\rm{TX}}}}}}}}}}-{\omega }_{{{{{{{{{\rm{LO}}}}}}}}}_{{{{{{{{\rm{RX}}}}}}}}}}| \pm {\omega }_{{{{{{{{\rm{IF}}}}}}}}}$. Thus, the desired sideband at IF can be brought down to baseband by setting the second stage LO frequency at the receiver to one of the center frequencies and depending on the frequency the baseband spectrum shall be conjugated or not. Notice that ${\omega }_{{{{{{{{{\rm{LO}}}}}}}}}_{{{{{{{{\rm{TX}}}}}}}}}}$ and ${\omega }_{{{{{{{{{\rm{LO}}}}}}}}}_{{{{{{{{\rm{RX}}}}}}}}}}$ have to be chosen such that the two IF bands are sufficiently separated.

### Link budget analysis

The power budget of the prototype was derived through a link budget analysis, which accounted for the path loss due to spreading, absorption, conversion loss of the mixer, and other cable, connector losses, the gain of antennas at the TX and RX, low noise amplifier, IF amplifier at the receiver. The received power after the first down-conversion stage is given by

4 $${P}_{{{{{{{{\rm{RX}}}}}}}}}={P}_{{{{{{{{\rm{TX}}}}}}}}}+\,{G}_{{{{{{{{\rm{TX}}}}}}}}}+\,{G}_{{{{{{{{\rm{RX}}}}}}}}}+\,{G}_{{{{{{{{\rm{LNA}}}}}}}}}\,-{L}_{{{{{{{{\rm{spread}}}}}}}}}-\,{L}_{{{{{{{{\rm{abs}}}}}}}}}-\,{L}_{{{{{{{{\rm{mixer}}}}}}}}}-\,{L}_{{{{{{{{\rm{misc}}}}}}}}}.$$

In this case, we considered 139 GHz and 224 GHz as the center frequencies for the LB and UB systems, respectively. The transmit power (*P*_TX_) is 13 dBm (LB) and 3 dBm (UB) at the corresponding RF frequency range. The antennas (*G*_TX_, *G*_RX_) utilized by the transceiver have 38 dBi gain for LB and 40 dBi gain for UB. The path loss, accounting for spreading (*L*_spread_) and absorption (*L*_abs_) loss, is obtained by considering 55% of humidity, 293 K temperature, and pressure of 1 atm, and is 108.1 dB for 139 GHz and 112.4 dB for 224 GHz (according the ITU model, as discussed next). The cable and connector loss (*L*_misc_) is 9.4 dB approximately for both setups. At the receiver, the loss incorporated due to a down-conversion mixer (*L*_mixer_) is around 7 dB and 15 dB for the LB and UB systems, correspondingly. The maximum Low Noise Amplifier (LNA) gain (*G*_LNA_) is about 12 dB (LB receiver) and 35 dB (UB receiver), which assists in utilizing the full dynamic range of the ADC.

Following the calculation, the theoretical received power (*P*_RX_) is −23.5 dBm and −19 dBm for 139 GHz system and 224 GHz, respectively, which closely match the received value of −21 dB (139 GHz) and −23 dB (224 GHz) for a 43-m-long LOS wireless link obtained in our experiments. These results support our system design and guide our PHY design.

### Experimental procedures

We deployed the equipment on the roof of two multi-story buildings (Egan Research Center and Snell Engineering) in the Northeastern University campus, with a distance of 43 m between the transmitter and receiver front-ends, as shown in Fig. 1. The experiments were run over multiple days, with cloudy weather, temperature between 9.9 °C and 13.6 °C, and humidity between 46% and 57.8%.

Before running our experimental campaign, the NI mmWave back-ends were properly calibrated to compensate imbalance between the in-phase and quadrature signal, as well as the DC offset. Additionally, the directional antennas of the analog front-ends were carefully aligned, to ensure that maximum antenna gain is experienced in both frequency bands. The alignment was performed manually, by transmitting a narrowband tone with a Keysight M8196A Arbitrary Waveform Generator and measuring the received power with a Keysight DSOZ632A Digital Storage Oscilloscope. We considered the antennas as accurately aligned when the measured received power matched that predicted by the link budget (as previously discussed).

The outdoor operations were performed in compliance with an FCC experimental license. The performance of the switching mechanism was evaluated by artificially triggering the switch, so that operations during actual passes of the Aura satellite over the deployment location were avoided (even though the UB system was actually operated in a frequency band that does not generate harmful interference to Aura’s MLS).

### Performance characterization of the prototype system

The profiling of the dual-link backhaul prototype has included several metrics shown in Fig. 2, which are detailed here.

- Thermal noise: noise characterization is an essential step to determine the detection algorithm and required signal processing. The thermal noise in the receiving chain and the absorption noise introduced by water molecules within the channel are the main sources of noise. Furthermore, the power supply and the transmission chain introduce low-frequency noise, which further impacts the received signals. In Fig. 2a, the histogram of the measured noise samples is shown for 800 MHz of bandwidth for the LB and UB front-ends. In both cases, the noise follows a Gaussian distribution. For LB system, the approximated mean and variance is −0.02 mV and 16 nW, respectively. In contrast, the mean and variance for UB system is −0.02 mV and 188 nW, respectively. Therefore, the noise power for the UB system is 10 dB higher than for the LB one, which explains the lower throughput for the UB system, for a similar link budget. Indeed, taking into account a 43 m distance wireless link with low PHY Bit Error Rate (BER) (~10^−2^ without coding), the aforementioned difference for the noise floor of the two front-ends explains why the LB system supports up to 16-QAM, whereas the UB system embraces up to QPSK only.
- Phase noise: phase noise is a major concern in frequency-multiplied systems. Phase noise refers to rapid, short-term, random fluctuations in phase caused due to time-domain instability of the oscillator. The SSB phase noise in terms of dBc/Hz is shown in Fig. 2b for the both LB and UB front-ends. Despite the utilization of large multiplier chains in front-ends, the use of PSGs with very high stability results in low phase noise at RF. The noise power drops below −100 dBc/Hz at 0.5 MHz for the LB front-end, and 0.8 MHz for the UB front-end. However, there are spikes due to spurious power components in the phase noise plot for both systems.
- Throughput and switching duration: the throughput and switching time have been measured during the experiments by combining information from the NI mmWave back-end system and the switching framework. The NI back-end is based on a OFDM PHY^31^, with the time resources organized intro frames of 10 ms (as in 3GPP NR), themselves split into 50 slots lasting 200 μs, and the frequency resources configured with eight component carrier with 100 MHz each (for a total of 800 MHz of bandwidth). The OFDM subcarrier spacing is 75 kHz. Each slot carries a variable number of data codewords, according to the modulation and coding scheme selected. The channel coding for the NI back-end is based on Xilinx 3GPP Mixed Mode Turbo Encoders/Decoders^50^. The back-end measures the throughput as the sum of the data bytes from correctly decoded codewords over a frame interval of 10 ms. Therefore, the logging granularity of our throughput measurements is 10 ms. The switching duration is computed by logging the time instant *T*_*i*_, *i* ∈ {TX, RX} of a switch event at the transmitter and receiver. Then, ${T}_{{{{{{{{\rm{s}}}}}}}}}={\min }_{i\in \{{{{{{{{\rm{TX}}}}}}}},{{{{{{{\rm{RX}}}}}}}}\}}{T}_{i}$ can be considered as the start time of a switch event. The end of a switch event is identified by the time instant ${T}_{{{{{{{{\rm{e}}}}}}}}}\ge {\max }_{i\in \{{{{{{{{\rm{TX}}}}}}}},{{{{{{{\rm{RX}}}}}}}}\}}{T}_{i}$ at which the throughput returns within ± 3% of its average value (to account for channel and noise fluctuations). The switch duration is then Δ_s_ = *T*_e_ − *T*_s_.

### Path loss model

The path loss for the link budgets in the evaluations of this paper is based on the sum of energy loss due to molecular absorption, and spreading loss. The latter is given by the free space path loss, i.e., in dB scale,

5 $${L}_{{{{{{{{\rm{spread}}}}}}}}}(d,f)=92.45+20{\log }_{10}(d)+20{\log }_{10}(f),$$

with *f* and *d* the carrier frequency (in GHz) and the propagation distance (in km), respectively. For a satellite orbiting at height *H*, the distance *d* for an elevation angle *α* can be computed as $d=\sqrt{{({R}_{{{{{{{{\rm{E}}}}}}}}}\sin \alpha )}^{2}+2{R}_{{{{{{{{\rm{E}}}}}}}}}H+{H}^{2}}-{R}_{{{{{{{{\rm{E}}}}}}}}}\sin \alpha$, with the Earth radius *R*_E_ = 6371 km.

The absorption loss *L*_abs_ is given by the molecular absorption from atmospheric gasses, including oxygen and water vapor. This factor has been computed using the line-by-line method described in ref. ^41^, which sums the contributions from each oxygen and water vapor resonance line, together with factors such as the pressure-induced nitrogen attenuation above 100 GHz. Unless differently specified, for the atmospheric composition and conditions, we considered the global reference atmosphere from the ITU report^42^, which provides a reference for the temperature, pressure, and water-vapor density.

## Data Availability

The experimental measurements data collected for this paper is archived on the Northeastern Library Digital Repository Service at http://hdl.handle.net/2047/D20427338. The data contains the experimental traces collected during the deployment of the dual-band backhaul prototype.

## Code availability

The software for the switching framework and to process the measurement traces is open source and available at https://github.com/mychele/dynamic-spectrum-sharing-active-passive-above-100-ghz^51^. The scripts have been tested with python version 3.9.7. The switching framework will require an API key which can be obtained from the N2YO website^37^. Additional requirements are listed in the code repository.
